# Association between Statin Use and Balance in Older Adults

**DOI:** 10.3390/ijerph17134662

**Published:** 2020-06-29

**Authors:** Antoine Langeard, Kathia Saillant, Elisabeth Charlebois Cloutier, Mathieu Gayda, Frédéric Lesage, Anil Nigam, Louis Bherer, Sarah A. Fraser

**Affiliations:** 1 Research Center, Institut Universitaire de Gériatrie de Montréal, Montreal, QC H3W 1W5, Canada; saillant.kathia@gmail.com (K.S.); e.charleb@gmail.com (E.C.C.); louis.bherer@umontreal.ca (L.B.); sarah.fraser@uottawa.ca (S.A.F.); 2 Department of Medicine, University of Montreal, Montreal, QC H3T 1J4, Canada; mathieu.gayda@icm-mhi.org (M.G.); anil.nigam@icm-mhi.org (A.N.); 3 EPIC Center, Montreal Heart Institute and University of Montreal, Montreal, QC H1T 1N6, Canada; 4 Research Center, Montreal Heart Institute, Montreal, QC H1T 1N6, Canada; frederic.lesage@polymtl.ca; 5 Department of Electrical Engineering, Polytechnique Montreal, Montreal, QC H3T 1J4, Canada; 6 Interdisciplinary School of Health Sciences, University of Ottawa, Ottawa, ON K1N 6N5, Canada

**Keywords:** statin, static, balance, falls, cardiovascular

## Abstract

*Background:* Several medications have been associated with an increased risk of balance deficits and greater likelihood to sustain a fall, representing a large health and economic issue. Statins are regularly prescribed to prevent strokes and heart attacks, but their impact on balance is unknown. The aim of this paper was to determine whether statin use is associated with poorer balance performances in older adults. *Methods:* All participants, one group taking statins (n = 34), and the other group not taking statins (n = 31), completed a balance assessment with their eyes closed and their eyes opened on a MatScan Pressure Sensing Mat. Center of Pressure (CoP) velocity, peak-to-peak distance, and standard deviation were collected in both anteroposterior (AP) and mediolateral (ML) directions. Multiple linear regression analyses were performed for each balance outcome, testing the statin use status as a predictor and controlling for appropriate factors including participants characteristics, lipid profile, and cardiovascular disease. *Results:* After controlling for confounding factors, statin use significantly predicted both CoP ML-Amplitude (β = 0.638, *p* = 0.004) and ML-Velocity (β = 0.653, *p* = 0.002) in the eyes-opened condition. *Conclusions:* The present study detected a negative association between statin use and balance control in the ML direction, suggesting that caution should be taken when prescribing statins in older adults, as this could decrease ML stability and ultimately increase fall and fracture risks.

## 1. Introduction

Falls in older adults represent a significant health and economic issue [1]. In the United States, approximately one-third of persons over 60 years of age fall annually, sustaining significant expenses related to the care of fall-related injuries [2]. Falls are responsible for restricted mobility, one of the major health concerns directly related to one’s independence, leading to the adoption of a sedentary lifestyle, an underlying factor for various pathological conditions including obesity, diabetes, and heart diseases [3]. Most falls have been associated with postural or cognitive deficits and with some medications [4]. The decline in postural control with age has often been detected through computerized measurement of the position of the center of pressure (CoP) [5]. CoP displacement analysis has predictive value for subsequent falls and is considered the gold standard of balance measurement [6]. It is, therefore, necessary to determine the different factors affecting CoP displacement in older adults. 

Some drugs that are regularly prescribed to older adults can be responsible for poorer balance [7]. However, further studies are needed to determine if cardiovascular drugs, in particular lipid-lowering medications, affect balance [8]. The age-related progression on the cardiovascular disease continuum starts with long-term cardiovascular risk factors, including hyperlipidemia, leading to coronary heart disease (CHD), the leading cause of death worldwide [9]. The number of lipid-lowering drugs (mostly statins) prescribed to older adults is therefore rising, and currently, more than 40% of older adults take these medications to manage and prevent cardiovascular conditions [10]. Statins are known to be safe and well-tolerated; however, evidence suggests that their use could be related to poorer balance.

First, statins could affect balance by altering muscle functionality. While the relationship between lower limb neuromuscular function and balance quality is well known [11], whether statin use decreases muscle functionality is a question of debate. Numerous studies did not detect any evidence of adverse effects of statins on various indicators of muscle integrity, such as lower limb strength measured through the functional Sit-to-Stand test [12] or handgrip and isometric maximal knee extension strength [13]. However, in clinical practice, statin therapy’s association with muscle problems can concern between 10% and 25% of the treated patients [14]. Muscle complaints and markers of mild muscle injury have been reported in statin users [15]. Statin use has also been reported to be associated with a decline in leg muscle quality and strength [16]. Moreover, evidence of myopathic weakness in statin users that improved upon cessation of the treatment has been found [17]. The reason for this discrepancy in findings could lie in the vast variety of tests used across the protocols to evaluate the neuromuscular function: subjective self-reports, scales, questionnaires, functional tests, or more precise measures through dynamometers. Scott et al. (2009) explained that statin use could have an indirect effect on strength as a result of neural changes, and the neural implications may differ according to the test used to evaluate muscle performances [16].

Whether these possible declines in neuromuscular function translate into a higher fall risk is also debated. After a one-year follow-up of adults aged between 70 and 90 years, statin users did not experience more falls than non-users [12]. Statin use has even been associated with a lower risk of falls in a large longitudinal study [18]. In contrast, a study of 774 older adults suggested that statins may exacerbate fall risk associated with aging [16]. These mixed results highlight the need for studies evaluating the effect of statin use on objective and more precise balance measures. 

To our knowledge, despite the importance of static balance in daily life activities of older adults and despite the fact that objective balance evaluation through CoP analysis is easy, highly reliable, and effective in assessing postural control [19], only one study focused on the effect of statins on balance in older adults [12]. While no association of statin use with muscle strength or mobility was detected, dynamic leaning balance was significantly reduced in statin users. Yet, static balance was not affected by statin use. However, the static balance assessment did not distinguish balance performances in the different spatial directions, and the effects of statin use were controlled for age and general health only. The authors acknowledged that further studies controlling for associated co-morbidities were needed to highlight a possible adverse effect of statins on balance. Indeed, associated cardiovascular risk factors like hypertension, glucose intolerance, obesity, or smoking can also be responsible for poorer postural control during standing [20]. 

The aim of the present study was, therefore, to determine if statin use in older adults is associated with changes in static balance evaluated through computerized MatScan Pressure Sensing Mat (Model 3150, Tekscan Inc., South Boston, MA, USA) and detailed CoP analysis in both anteroposterior and mediolateral directions while controlling for associated cardiovascular and anthropometric risk factors. 

## 2. Materials and Methods 

### 2.1. Participants 

Participants 60 years old or older were recruited through the clinical practice of cardiac physicians and advertisements at the Institut Universitaire de Gériatrie de Montréal and the Preventive medicine and Physical activity center (Centre ÉPIC) of the Montreal Heart Institute (MHI). Participants did not have severe hearing impairment, uncorrected visual impairment, or cognitive impairment. They had to be able to walk 15 meters without the use of an assistive device or the assistance of another person. A research assistant collected anthropological data (age, sex, body mass index (BMI), education), cognitive abilities (Montreal Cognitive Assessment, MoCA) and data on depression status (Geriatric Depression Scale, GDS), Fear of Falling (Fall Efficiency Scale, FES), physical activity (Physical Activity Scale for the Elderly, PASE), and the presence of past falls in the 6 months prior to inclusion (self-reported). Enrolled participants were screened by a medical practitioner who assessed the presence of cardiovascular risk factors and associated diseases (tobacco use, hypertension, diabetes, coronary artery disease), conducted a blood lipidic profile analysis ( high-density lipoprotein (HDL)-cholesterol, triglycerides, and low-density lipoprotein (LDL)-cholesterol), and collected the number and the names of the prescribed drugs. A maximal exercise treadmill test with an individualized ramp protocol was also performed following a previously described protocol [21]. Maximal exercise tolerance was defined as the highest level of metabolic equivalents (METs). Resting systolic and diastolic blood pressure measurements were performed in a supine position before the test. 

Participants were grouped according to statin use: those not taking statins (no-statins group) and those taking statins (statins group). This study was approved by the Montreal Heart Institute ethics (Project #2012-264, 1386 – CARDIO-COGNIRS) and was performed in accordance with the ethical standards of the 1964 Declaration of Helsinki and its later amendments. Written informed consent was obtained from all individual participants included in the study.

### 2.2. Posturography Evaluation

All participants performed two 10-second static balance tests requiring them to stand on two feet barefoot on a MatScan pressure measurement system (Model 3150, Tekscan Inc., South Boston, MA, USA), first with their eyes opened and then with their eyes closed. Participants were asked to position their feet on the platform side by side, slightly apart, in a comfortable standing position. The MatScan system has demonstrated good to excellent reliability for anteroposterior (AP) and mediolateral (ML) sway, with eyes opened and closed during quiet stance on two limbs, making this tool valid to use in clinical and research settings [22]. CoP displacements were recorded, and three parameters reflecting postural stability were calculated in both AP and ML directions. One was a speed parameter (CoP-Velocity), one was an amplitude parameter (CoP peak-to-peak distance, CoP-ptp), and one was a variability parameter (CoP standard deviation, CoPsd). Higher scores of these parameters represent poorer postural control and are known to be related to postural instability implicated in the increased risk of falling [6,23]. 

### 2.3. Statistical Analyses

Characteristics of participants were compared according to statin use (users vs. non-users) using Mann–Whitney tests or chi-square tests (for binomial variables) on means and standard deviations for continuous variables or percentages for binomial variables. Multiple linear regression analyses were performed for each balance outcome, testing the statin use status as a predictor and controlling for the characteristics that differed between the groups. No evidence of multicollinearity or heteroscedasticity was found. Statistical analyses were performed using JASP Version 0.8.5.1 (JASP Team, Amsterdam, Netherlands) and an alpha of *p* = 0.05 was set for significance.

## 3. Results

Table 1 presents the characteristics of the two groups. In comparison with non-users, participants taking statins were significantly older and were more likely to be men and to have a higher BMI. They were also more likely to be diagnosed with hypertension, be taking more drugs, and have lower levels of HDL- and LDL-cholesterol. Age, sex, BMI, METS, hypertension, coronary artery disease (CAD), number of drugs, HDL-cholesterol, and LDL-cholesterol were therefore computed as covariates in the regression analysis in order to determine the statin effect while controlling for these associated factors. The balance performance values for the two groups are presented in Table 2. The results of the regression analyses are presented in Table 3. Three significant regression equations based on statin use and the different covariates (age, sex, BMI, METS, hypertension, CAD, number of drugs, HDL-cholesterol, and LDL-cholesterol) were found predicting eyes-opened ML-amplitude, eyes-opened ML-velocity, and eyes-closed AP-variability. The unstandardized coefficients of these equations are presented in Table 4.

**Eyes-opened ML-Amplitude:** A significant regression equation was found predicting eyes-opened ML-Amplitude (*F* (1,53) = 2.359, *p* = 0.022) with an R^2^ of 31.2% (Table 3). After adjusting for covariates, statin use predicted CoP ML-Amplitude (β = 0.609, *p* = 0.006). Participants taking statins had an ML-Amplitude of 0.537 cm, higher than that of those who were not taking statins (95% CI [0.161–0.914]). None of the covariates was significant in the model (Table 4). 

**Eyes-opened ML-Velocity:** A significant regression equation was also found predicting eyes-opened ML-Velocity (*F* (1,53) = 3.168, *p* = 0.003) with an R^2^ of 37.9% (Table 3). After adjusting for covariates, statin use remained a significant predictor (β = 0.627, *p* = 0.003). Participants taking statins had a velocity of 0.221 cm·s^−1^, higher than that of those who were not taking statins (95% CI [0.078–0.364]). Among the covariates, BMI and LDL-cholesterol also significantly influenced the model (β = −0.339, *p* = 0.039 and β = 0.414, *p* = 0.010, respectively) (Table 4). 

**Eyes-closed AP-Variability:** A significant regression equation was found predicting eyes-closed AP-Variability (*F* (1,53) = 2.129, *p* = 0.038) with an R^2^ of 29.0% (Table 3). However, statin use was not a significant predictor (Table 4).

## 4. Discussion

The aim of the present study was to evaluate the association between statin use and static balance in a sample of older adults while controlling for confounding factors that might influence bipedal balance. This study is the first to reveal that statin use is associated with poorer balance performance, in particular in the mediolateral direction. Statin use was associated with an increase of 0.23 cm·s^−1^ of the ML-Velocity of the CoP and an increase of 0.563 mm of the ML-Amplitude of the CoP while controlling for associated comorbidities. These coefficients are very close to the ML sway differences between non-fallers and recurrent fallers observed by Brauer et al. (2000) (0.2 and 0.5, respectively) [24]. This highlights the clinical significance of the present findings, especially since Brauer et al. [24] and Maki et al. [25] have determined that, even during a minimally challenging task, it is the specific reduction in ML control that is associated with an increased risk of falling. 

These results are in contrast to the findings of Haerer et al. (2012), which showed no effect of statin use on quiet stance [12]. One reason for the discrepancy between the present study and that of Haerer et al. [12] is related to the balance performance measures and how they were assessed in the two studies. Haerer et al. did not assess motion in specific directions but only as a global area [12]. This could have masked the specific effects in the ML direction found in the present study. Further, there were several significant differences between those taking statins and those not taking statins (e.g., lipidic profile, hypertension, etc.) recorded and controlled for in the present study that were not controlled for by Haerer et al. [12].

A possible explanation for the effect of statins on balance could come from their deleterious effects on muscle functions [14,15,16,17]. Statin use has been shown to be responsible for reduced citrate synthase, possibly disrupting mitochondrial respiration and activating pathways of apoptosis or autophagy in muscle fibers [26]. Of interest, in quiet standing with feet side by side, ML balance is predominantly controlled by the hip muscles (abductors/adductors) [27]. While the effect of statin use on these specific muscle groups is unknown, it can be hypothesized that hip muscles may have been affected in the statin users, resulting in poorer ML balance. This could also explain why Haerer et al. [12] found significant differences based on statin use in the leaning balance assessment and not in the standing balance assessment. A more active balance condition may help delineate differences based on statin use.

Whether the present results on balance actually translate to a higher number of falls could not be investigated in the present study. Retrospective self-report in older adults, as recorded in the present study, often results in misestimation of the true incidence of falls [28]. Moreover, statin-associated memory loss has been previously reported and could lower the number of retrospective self-reported falls in the participants taking statins [29]. This could possibly explain the surprisingly lower number of falls reported by statin users in the present study. Moreover, more information on the type of falls and longitudinal follow-up of the number of falls would be needed to determine whether a causal relationship exists between statin use and falls in older adults.

However, one of the strengths of the present study is the use of an objective measure of balance through computerized analysis of CoP displacement, a method known to overcome the drawbacks of functional tests as it is less variable across examiners, more sensitive to changes, and therefore particularly appropriate in order to evaluate an intervention’s effects [30]. Another strength is the control for numerous confounders, including the lipid profile and the presence of associated diseases in the participants, which was a drawback of past studies like the only other studies on the effect of statin use on balance, which controlled only for age and general health status [12].

The present study also presents a few limits. Investigating the effects of different statin medications could not be performed, given the relatively small sample; however, Scott et al. (2009) reported no significant differences between the types of statin administered when assessing effects of statin use on strength or fall rates [16]. Duration of treatment and dose effects should also be further studied in future larger samples. Unmeasured confounders could also be partly responsible for our results, such as a healthier lifestyle, the socioeconomic status, as well as other drugs that could have more influence on balance control, like psychotropic medications [8]. Moreover, while the prevalence of diabetes was not statistically different between participants taking statins and those not taking statins, it could still have impacted the present results, in particular if participants in the statin group had a longer duration of diabetes and poorer glucose control [30]. Indeed, the participants of the present study were not randomized to statin use, and like all observational studies of pharmacologic exposures, confounding by indication could have impacted the results. Given the relatively small number of participants in each group, controlling for the confounding factors may not have been optimal, and randomized controlled trials are needed to confirm the present findings and further identify the mechanisms that underlie the relationship between statins and balance in older adults.

## 5. Conclusions

Statins have been proven to be safe and efficient in reducing coronary heart disease, strokes, and mortality [31]. Reducing multiple medication use and its association with important deleterious effects on both cognition and mobility [32] could be possible, but assessing the relative merit and the probabilities of benefit and harm of specific medications is needed [33]. The present results suggest a negative effect of statins on balance control in the mediolateral direction, a direction that has been associated with increased falls risk. Particular attention should be given to the risk of balance loss in older adults using statins. Monitoring balance quality during the treatment could be of interest in order to detect patients at risk. The results suggest that statin use may worsen balance abilities; if these results are confirmed, they could be taken into account in assessing the benefits/risks ratio when initiating treatment and, when possible, alternative therapies like physical activity or dietetic interventions could be implemented.

## Figures and Tables

**Table 1:** Participants characteristics (means ± standard deviations for continuous variables or percentages for binomial variables).

	No Statin Use (n = 34)	Statin Use (n = 31)	*p*
Age (years)	66.67 ± 5.46	70.23 ± 5.00	0.008 *
Sex (% of men)	41.2%	67.7%	0.032 *
BMI (kg·m^−2^)	26.55 ± 5.42	30.09 ± 5.14	0.003 *
Education (years)	15.38 ± 4.20	16.61 ± 3.91	0.288
MoCA	27.21 ± 2.31	26.52 ± 2.32	0.19
GDS	4.91 ± 4.17	3.43 ± 3.59	0.18
FES	20.00 ± 3.87	20.32 ± 3.00	0.296
PASE	124.99 ± 69.50	132.35 ± 64.98	0.686
METS	9.31 ± 2.23	7.98 ± 1.94	0.013 *
SBP (mmHg)	127.85 ± 11.17	129.19 ± 15.87	0.693
DBP (mmHg)	76.47 ± 6.23	76.42 ± 8.75	0.978
Tobacco use	2.9%	9.7%	0.254
Hypertension	32.4%	61.3%	0.019 *
CAD	9.1%	48.4%	<0.001 *
Diabetes	8.8%	25.8%	0.068
Number of drugs	0.44 ± 0.79	3.13 ± 1.18	<0.001 *
HDL-cholesterol (g·L^−1^)	1.56 ± 0.51	1.24 ± 0.26	0.004 *
Triglycerides (g·L^−1^)	1.19 ± 0.61	1.41 ± 0.70	0.174
LDL- cholesterol (g·L^−1^)	3.09 ± 0.86	1.98 ± 0.65	<0.001 *
Self-reported falls in the past 6 months	8/34	3/31	0.123

BMI: body mass index, MoCA: Montreal Cognitive Assessment, GDS: Geriatric Depression Scale, FES: Fall Efficiency Scale, PASE: Physical Activity Scale for the Elderly, MET: Metabolic Equivalent of Task, SBP: systolic blood pressure, DBP: Diastolic blood pressure, Hypertension: received a diagnosis of hypertension, CAD: coronary artery disease, HDL-cholesterol: High-Density Lipoproteins, LDL-cholesterol: Low-Density Lipoproteins. * *p* < 0.05.

**Table 2:** Balance performances according to statin use (means ± standard deviations).

			No Statin Use (n = 34)	Statin Use (n = 31)
EYES OPENED	AP	Amplitude (cm)	1.166 ± 0.415	1.326 ± 0.504
		Velocity (cm·s^−1^)	0.670 ± 0.207	0.759 ± 0.199
		Variability (cm)	0.336 ± 0.120	0.373 ± 0.157
	ML	Amplitude (cm)	0.811 ± 0.340	1.113 ± 0.489
		Velocity (cm·s^−1^)	0.728 ± 0.194	0.764 ± 0.176
		Variability (cm)	0.217 ± 0.092	0.299 ± 0.142
EYES CLOSED	AP	Amplitude (cm)	1.564 ± 0.610	1.885 ± 0.563
		Velocity (cm·s^−1^)	0.896 ± 0.368	1.293 ± 0.708
		Variability (cm)	0.433 ± 0.168	0.524 ± 0.182
	ML	Amplitude (cm)	0.954 ± 0.504	1.272 ± 0.584
		Velocity (cm·s^−1^)	0.822 ± 0.246	0.975 ± 0.355
		Variability (cm)	0.243 ± 0.101	0.333 ± 0.162

ML: mediolateral, AP: Anteroposterior.

**Table 3:** Characteristics of the multiple regression models according to the predicted balance outcomes.

			F (1.53)	*p*	R^2^
EYES OPENED	AP	Amplitude (cm)	1.447	0.187	0.218
		Velocity (cm·s^−1^)	1.502	0.165	0.224
		Variability (cm)	1.712	0.102	0.248
	ML	Amplitude (cm)	2.359	0.022	**0.312**
		Velocity (cm·s^−1^)	3.168	0.003	**0.379**
		Variability (cm)	1.853	0.074	0.263
EYES CLOSED	AP	Amplitude (cm)	1.831	0.078	0.260
		Velocity (cm·s^−1^)	1.247	0.285	0.193
		Variability (cm)	2.129	0.038	**0.290**
	ML	Amplitude (cm)	1,388	0.212	0.211
		Velocity (cm·s^−1^)	1,215	0.303	0.189
		Variability (cm)	1,335	0.237	0.204

Significant findings in bold.

**Table 4:** Coefficient of the predictors in the significant regression models.

	Eyes Opened ML Velocity (cm·s^−1^)	Eyes Opened ML Amplitude (cm)	Eyes Closed AP Variability (cm)
Statin	0.221 (0.071) **	0.537 (0.187) **	0.039 (0.078)
Age (years)	0.005 (0.005) *	0.024 (0.013)	0.007 (0.006)
Sex (% of men)	0.038 (0.062)	0.022 (0.163)	0.116 (0.068)
BMI (kg·m^−2^)	−0.011 (0.005) **	0.002 (0.014)	0.003 (0.006)
METS	0.015 (0.016)	0.042 (0.043)	−0.015 (0.018)
Hypertension	0.061 (0.052)	0.202 (0.137)	0.053 (0.057)
CAD	0.043 (0.065)	0.259 (0.171)	0.048 (0.071)
Number of drugs	−0.042 (0.029)	−0.143 (0.075)	−0.008 (0.031)
HDL- cholesterol (g·L^−1^)	−0.060 (0.063)	0.067 (0.165)	−0.057 (0.069)
LDL- cholesterol (g·L^−1^)	0.077 (0.029) **	0.016 (0.076)	0.062 (0.032)

*, ** significant predictor.
